# Special Issue “Role of STAT3 in Oncogenesis”

**DOI:** 10.3390/biomedicines10112689

**Published:** 2022-10-24

**Authors:** Kwang Seok Ahn

**Affiliations:** Department of Science in Korean Medicine, Kyung Hee University, 24 Kyungheedae-ro, Dongdaemun-gu, Seoul 02447, Korea; ksahn@khu.ac.kr; Tel.: +82-2-961-2316

This *Biomedicines* Special Issue was designed to attract articles that focused on the pleiotropic role of the signal transducer and activator of transcription 3 (STAT3) transcription factor in different facets of tumorigenesis. STAT3 belongs to the signal transducer and activator of transcription (STAT) family. Upon activation by various cytokines and growth factors, such as IL-6, epidermal growth factor (EGF) and platelet-derived growth factor (PDGF) [1,2], STAT3 undergoes tyrosine (Tyr705) phosphorylation to regulate its dimerization via phosphortyrosine-SH2 domain interactions. This allows STAT3 nuclear translocation and DNA binding to induce transcription. The tyrosine phosphorylation is commonly mediated by the Janus Kinase (JAKs), particularly JAK2. Cytokine-induced STAT3 activation is tightly controlled and regulated by SHP phosphatases and suppressor of cytokine signaling 3 (SOCS3), which can inhibit STAT3 activation [3,4]. However, in cancer cells, STAT3 are often constitutively activated, leading to high expression of different STAT3-related genes involved in the survival, metastasis, angiogenesis and immune suppression [5]. Hence, there is increased interest in the suppression of STAT3 signaling as one of the therapeutic strategies against cancer. Thus, this Special Issue was prepared to contain both review articles as well as novel research studies that offered insights into novel mechanisms regulating STAT3 activation in tumor cells, its potential interaction with other signaling molecules and innovative strategies to target its aberrant activation.

Byun W.S. and colleagues [6] focused on the fact that triple-negative breast cancer (TNBC) is resistant to docetaxel and suggested that pulvomycin, isolated from marine-derived actinomycete, is a new anti-cancer strategy in docetaxel-resistant TNBC. The authors established docetaxel-resistant MDA-MB-231 cells (MDA-MB-231-DTR) and confirmed that p-STAT3 (Y705) activity was increased in the cell line. The treatment with pulvomycin in MDA-MB-231-DTR suppressed STAT3 activation, induced arrest of the G0/G1 cell cycle and induced apoptotic proteins, such as the cleaved form of PARP and caspase-3. In addition, the co-treatment with docetaxel and pulvomycin effectively attenuated tumor growth through STAT3 downregulation in MDA-MB-231-DTR-bearing transgenic mouse model. Based on these findings, the authors suggest that pulvomycin, a new STAT3 inhibitor, is an effective strategy for overcoming docetaxel-resistant TNBC. 

Yang M.H. et al. [7] analyzed the anti-neoplastic actions of Albendazole (ABZ) against gastric cancer cells. In gastric cancer, the p-STAT3 and p-STAT5 proteins have abnormal overexpression, which is the result of cancer growth and progression. The authors found that ABZ suppressed the aberrant expression of p-STAT3 and p-STAT5 in gastric cancer cell lines. They also reported that ABZ suppressed JAK and Src proteins, which are upstream signaling kinases of STAT family proteins. In addition, ABZ induced the SHP-1 protein, which can act to dephosphorylate STAT family proteins. According to the authors, ABZ altered the levels of various oncogenic and apoptotic proteins and induced ROS to influence the STAT signaling pathway. Treatment with ABZ after pre-treatment with NAC, a ROS inhibitor, substantially alleviated ABZ’s anti-cancer effect. Based on these results, the authors suggested that, as an anti-tumor agent, ABZ inhibited STAT3 and STAT5 activation and upregulated the SHP-1 protein through induction of ROS accumulation.

Bauvois B. and colleagues [8] discovered that the triggering of interferon (IFN) receptors had a positive effect on the survival of chronic lymphocytic leukemia (CLL) patients and studied the interaction between IFN and CLL cells. These authors confirmed that one of the survival processes, the STAT3 signaling pathway, was affected when CLL cells were treated with type I and II IFNs. Type I and II IFNs increased mitochondrial activity in CLL and were involved in the intrinsic apoptotic pathway. In addition, IFNs affected CLL survival via the STAT3/Mcl-1 signaling pathway. In this process, JAK2, Src and Tyk2 tyrosine kinase were involved in the activation of STAT3 molecules. Based on these results, the authors found that type I and II IFNs activated the STAT3/Mcl-1 signaling pathway in primary CLL cells via Tyk2 and Src or JAK2 and Src kinases. 

Gonnella R. et al. [9] described that the highly aggressive B-cell lymphoma, primary effusion lymphoma (PEL), has unfolded protein response (UPR) activity and sustained activation of the oncogenic pathways, such as the STAT3 pathway. It was found that the inositol-requiring kinase (IRE) 1α/X-box binding protein (XBP1) axis of the UPR plays a very important role in the survival of PEL cells. The authors showed that inhibition of the IRE1α/XBP1 axis by 4µ8C reduced PEL cells’ survival, and this inhibition also reduced the release of pro-inflammatory/immunosuppressive cytokines, such as IL-6, IL-10 and VEGF, and downregulated the activity of STAT3. The authors also confirmed the effect on autophagy, which is an inhibitory effect on intracellular UPR. As a result, it was shown that inhibition of the IRE1α/XBP1 axis promoted an autophagy flux. In addition, the authors found that silencing the *XBP1* gene increased autophagy and decreased cell viability. Based on these results, the authors argued that targeting the UPR, especially the IRE1α/XBP1 axis, is a very prospective strategy for the anti-cancer effect of PEL. 

Hong S.J. and colleagues [10] examined the anti-cancer effects of the electrophilic Deguelin analog by inhibiting STAT3 signaling in H-*RAS*-transformed human mammary epithelial cells. The authors investigated which of the nine Deguelin analogs inhibited the STAT3 factor in H-*Ras*-transformed human cells. Among these substances, they proposed that factor SH48 with α, β-unsaturated carbonyl moiety suppressed the dimerization and translocation of STAT3 into the nucleus. In addition, SH48 was formed in a complex with STAT3. It was demonstrated that SH48 suppressed the expression of p-STAT3 (Y705), did not cause apoptosis but induced autophagic cell death. Based on these findings, the authors argued that the cysteine 259 residue of STAT3 is a potential target site of STAT3 and that substances with α, β-unsaturated carbonyl moiety may form a complex with STAT3 by binding the cysteine 259 residue. 

Pham T.H. et al. [11] investigated the interaction between STAT3 and p53 and reviewed the dual-targeted therapies. They described the role of STAT3 and how STAT3 acts as an oncogene in cancer. They also explained six strategies that suppressed STAT3 signaling in cancer: (1) Suppressing STAT3 upstream proteins, (2) Targeting the SH2 domain of STAT3, (3) Blocking STAT3 DNA-binding sites, (4) Inhibiting the STAT3 N-terminal domain, (5) Suppressing *STAT3* mRNA expression, (6) Targeting STAT3 endogenous negative regulators. They also reviewed the features of p53 and described how p53 mutation caused cancer formation. They mentioned two strategies targeting mutant p53: (1) a strategy to accumulate the gain-of-function p53 (GOF p53) to inactivate mtp53 and restore it to wild type, (2) a strategy to block the generation of wild-type p53 (wtp53), which can break the interaction between wtp53 and MDM2/MDM4, a negative regulator of p53. The authors explained the functions and roles of STAT3 and p53 and how they were related to each other. STAT3 inhibited p53-mediated apoptosis and sustained STAT3 activation induced p53 mutations. Based on these results, the authors argue that inhibitors targeting STAT3-p53 are very potential dual-target agents for anti-cancer therapy. This review article describes the function of STAT3 and p53 in cancer and drugs targeting these two factors. 
